# Biodegradable Nanocomposites Based on Blends of Poly(Butylene Adipate–Co–Terephthalate) (PBAT) and Thermoplastic Starch Filled with Montmorillonite (MMT): Physico-Mechanical Properties

**DOI:** 10.3390/ma17030540

**Published:** 2024-01-23

**Authors:** Hamed Peidayesh, Leoš Ondriš, Simona Saparová, Mária Kovaľaková, Oľga Fričová, Ivan Chodák

**Affiliations:** 1 Polymer Institute of the Slovak Academy of Sciences, Dúbravská Cesta 9, 845 41 Bratislava, Slovakia; hamed.peidayesh@savba.sk; 2 Department of Physics, Faculty of Electrical Engineering and Informatics, Technical University of Košice, Park Komenského 2, 042 00 Košice, Slovakiasimona.saparova@tuke.sk (S.S.); maria.kovalakova@tuke.sk (M.K.); olga.fricova@tuke.sk (O.F.)

**Keywords:** thermoplastic starch, poly(butylene adipate–co–terephthalate), montmorillonite, melt blending process, nanocomposites

## Abstract

Poly(butylene adipate–co–terephthalate) (PBAT) is widely used for production of biodegradable films due to its high elongation, excellent flexibility, and good processability properties. An effective way to develop more accessible PBAT-based bioplastics for wide application in packaging is blending of PBAT with thermoplastic starch (TPS) since PBAT is costly with prices approximately double or even triple the prices of traditional plastics like polyethylene. This study is focused on investigating the influence of TPS/PBAT blend ratio and montmorillonite (MMT) content on the physical and mechanical properties and molecular mobility of TPS–MMT/PBAT nanocomposites. Obtained TPS–MMT/PBAT nanocomposites through the melt blending process were characterized using tensile testing, dynamic mechanical thermal analysis (DMTA), and X-ray diffraction (XRD), as well as solid-state ^1^H and ^13^C NMR spectroscopy. Mechanical properties demonstrated that the addition of TPS to PBAT leads to a substantial decrease in the tensile strength as well as in the elongation at break, while Young’s modulus is rising substantially, while the effect of the MMT addition is almost negligible on the tensile stress of the blends. DMTA results confirmed the formation of TPS domains in the PBAT matrix. With increasing TPS content, mobility of starch-rich regions of TPS domains slightly increases. However, molecular mobility in glycerol-rich regions of TPS domains in the blends was slightly restricted. Moreover, the data obtained from ^13^C CP/MAS NMR spectra indicated that the presence of TPS in the sample decreases the mobility of the PBAT chains, mainly those located at the TPS/PBAT interfaces.

## 1. Introduction

Biodegradable plastics from renewable resources have attracted much attention in the last two decades due to increasing concern about the serious negative effects of fossil-based plastics on the ecological environment [1]. Starch has been considered as one of the most promising biodegradable polymers owing to its abundant availability, low cost, and production from renewable resources [2,3,4]. It is necessary to make the starch processable using standard techniques since native starch degrades before reaching melting point, which is well above 200 °C, due to strong intermolecular hydrogen bonds [5]. Therefore, thermoplastic starch (TPS) is obtained by the incorporation of plasticizers such as glycerol, sorbitol, urea, etc., at higher temperatures, applying shear force [6,7,8,9]. However, TPS exhibits poor mechanical properties caused mainly due to moisture absorption related to the hydrophilic nature of starch and recrystallization phenomena which are observed after a longer time of storing [10].

Many strategies have been developed to improve the performance of starch-based materials [11,12]. As mentioned above, TPS suffers from retrogradation and recrystallization. An effective approach to improve the mechanical properties consists of blending TPS with hydrophobic biodegradable plastics such as polyhydroxyalkanoate (PHA) [13,14], poly(lactic acid) (PLA) [15,16], poly(ε–caprolactone) (PCL) [17,18], and poly(butylene adipate–co–terephthalate) (PBAT) [19,20,21,22]. Among all of these polymers which are mixed with TPS, a biodegradable copolyester, PBAT, has attracted great attention since its physical and mechanical properties are similar to those of polyethylene [23,24]. However, the poor compatibility between the hydrophilic TPS and hydrophobic PBAT makes the PBAT/TPS blends not so easy to prepare, and especially, their application properties are changing in many cases [25]. Interestingly, some studies reported that inorganic fillers such as sodium montmorillonite (NaMMT) and zeolite could enhance the compatibility between TPS and hydrophobic plastics such as PLA [26] and PE [27], which avoids some of the drawbacks of using compatibilizers containing maleic anhydride due to its potential toxicity.

On the other hand, another common approach to increase the mechanical strength and stiffness is the addition of reinforcing nanoparticles [28,29,30]. In this regard, reinforcing fillers not only could enhance the mechanical properties but also act as compatibilizers between the polymers. The most recent research is focused on montmorillonite (MMT) due to its well-controlled chemical properties, availability, and versatility towards the environment and health. MMT, a layered silicate, forms an intercalated structure by penetration of large organic molecules inside the interlayer space of the nanofiller forming so-called organomodified nanoparticles [31]. During further processing, applying shear stress, the organomodified MMT particles decompose to individual layers with an extremely high surface area [8,32,33] which can be dispersed in the TPS matrix independently, forming exfoliated MMT nanoparticles [34]. A high surface area is the basis for the extensive changes in physical properties. The incorporation of inorganic fillers such as MMT and sepiolite nanoclays into TPS/PBAT blends has been reported in the literature [35,36]. However, these studies have investigated only the tensile and thermal properties of the nanocomposites. To the best of our knowledge, there are no reports on investigating the physical and mechanical properties, structure, and molecular mobility of nanocomposites based on blends of PBAT and TPS filled with MMT. By appropriate blending of PBAT with TPS, a material with desired properties and a competitive price can be achieved [37].

The main goal of the present work was to evaluate the influence of the TPS/PBAT blend ratio and MMT content on the physical and mechanical properties and molecular mobility of TPS–MMT/PBAT nanocomposites. In the context of this work, the TPS–MMT/PBAT nanocomposites were characterized using tensile testing, dynamic mechanical thermal analysis (DMTA), and X-ray diffraction (XRD), as well as solid-state ^1^H and ^13^C NMR spectroscopy. The aim of this publication is to obtain general information on possibilities to modify properties of the PBAT/TPS mixture by modification of the TPS. The hypothesis assumes that if one of the major components will improve its ultimate properties, this should also positively affect the performance of the final blend. Since the tensile strength of the PBAT/TPS mixture is decreasing compared to the virgin PBAT material, the introduction of reinforcing fillers to the TPS should contribute to higher strength and, possibly, also to other parameters, e.g., water/moisture uptake and stability of the properties during storage. Moreover, the addition of TPS as an inexpensive biodegradable component results in a substantial decrease in the price of the final mixture, making the biodegradable blend economically more competitive compared to, e.g., polyethylene, in spite of the necessity to accept a certain decrease in the ultimate properties of the product.

## 2. Materials and Methods

### 2.1. Materials

Native corn starch Meritena^®^ 100 was provided by Brenntag (Bratislava, Slovakia). Its water content was around 12 wt% as determined by drying in the oven at 100 °C for 5 h. Poly(butylene adipate–co–terephthalate) (PBAT), grade Ecoworld™ 003 with MFI < 5 (according to DIN ISO 1133 [38] at 190 °C and 2.16 kg), was supplied by Jinhui Zhaolong Co. (Shanxi Xiaoyi, China). Natrified montmorillonite (Cloisite^®^ Na^+^) with a cationic exchange capacity range of 80–95 mequiv/100 g was purchased from Southern Clay Products (Gonzales, TX, USA). Glycerol was obtained from Centralchem, Ltd. (Bratislava, Slovakia). Double distilled water was used for the preparation of all solutions.

### 2.2. Preparation of TPS–MMT/PBAT Nanocomposites

First, TPS–MMT samples were prepared as follows. Cloisite Na (MMT) particles at content of 2 and 5 php (parts per 100 parts of polymer, based on the dry weight of starch) were dispersed in a mixture of water and glycerol by mechanical mixing at ambient temperature for 5 min. The suspension of starch, MMT, glycerol, and water was continuously mechanically stirred at 80 °C for 10 min. The weight ratio of starch/glycerol/water was 1.0:0.5:2.3. Then, the suspension was dried in the oven at 100 °C for 5 h, and it was left overnight at 60 °C to prevent moisture absorption. Then, TPS–MMT materials (containing MMT when appropriate) were processed in a laboratory mixer Plastograph Brabender PLE 331 for 10 min at 130 °C and 100 rpm (rounds per minute). Afterward, the obtained TPS–MMT materials were blended with PBAT in different ratios 10/90, 20/80, and 30/70 in another chamber for 10 min at 130 °C and 100 rpm. The slabs around 1 mm thick were prepared through compression molding (laboratory press, Fontijne TP 50, Delft, The Netherlands) at 130 °C using 2 min preheating without pressure and an additional 2 min at a pressure of 2.65 MPa. The obtained samples were stored in airtight plastic containers for 48 h at ambient conditions before performing characterizations.

### 2.3. Mechanical Properties

The tensile properties of the nanocomposites were measured at room temperature using an Instron 3365 universal testing machine (Instron, Norwood, MA, USA) at cross-head speed of 50 mm·min^−1^ in uniaxial deformation. According to the standard ASTM D638 [39], the dog-bone testing specimens with the dimensions of the deformed area 3.5 × 30 mm^2^ and thickness of approximately 1 mm (exactly measured by micrometer before testing) were used. The mean values and standard deviations were calculated from seven specimens for all parameters.

### 2.4. Dynamic Mechanical Thermal Analysis (DMTA)

DMA measurements of the nanocomposites were carried out using a DMA Q800 (TA Instruments, New Castle, DE, USA). The samples (ca. 30 × 6 × 1 mm^3^) were measured at a frequency of 5 Hz and an amplitude of 5 μm in tensile mode with a heating rate of 2 °C·min^−1^.

### 2.5. X-ray Diffraction (XRD)

XRD measurements were taken on a MiniFlex600 XRD analyzer (Rigaku, Tokyo, Japan). The XRD was performed using the theta–2theta method at a voltage of 40 kV and a current of 15 mA. Cu Kα X-rays with wavelength λ = 0.154 nm were used. The sample and the detector rotated at 2.5°/min and 5°/min, respectively.

### 2.6. Nuclear Magnetic Resonance (NMR)

The samples were cut into small pieces and filled into 4 mm ZrO_2_ rotors. NMR spectra were acquired on a 400 MHz Varian spectrometer (Palo Alto, CA, USA) at room temperature. ^1^H NMR spectra were performed on static samples as well as using the magic angle spinning technique (MAS NMR spectra) with a MAS rate of 10 kHz. The π/2 pulse duration was 2.9 μs, the spectra were detected with a delay of 10 s and acquisition time of 20 ms, and 32 scans were accumulated for the final spectra. ^1^H BL NMR spectra were measured using Chen’s method [40]. ^13^C CP/MAS NMR measurements were performed using cross-polarization (CP) and MAS techniques simultaneously with a MAS rate of 10 kHz. Cross-polarization contact time was 2 ms, acquisition time 40 ms, power heteronuclear decoupling 52 kHz, delay time was 10 s, and 10,000 scans were accumulated. Adamantane was used for the calibration of ^1^H and ^13^C chemical shifts. The measured spectra were processed using Mestrelab Research Mnova 9.0 software.

## 3. Results and Discussion

### 3.1. X-ray Diffraction (XRD)

**Figure 1:**
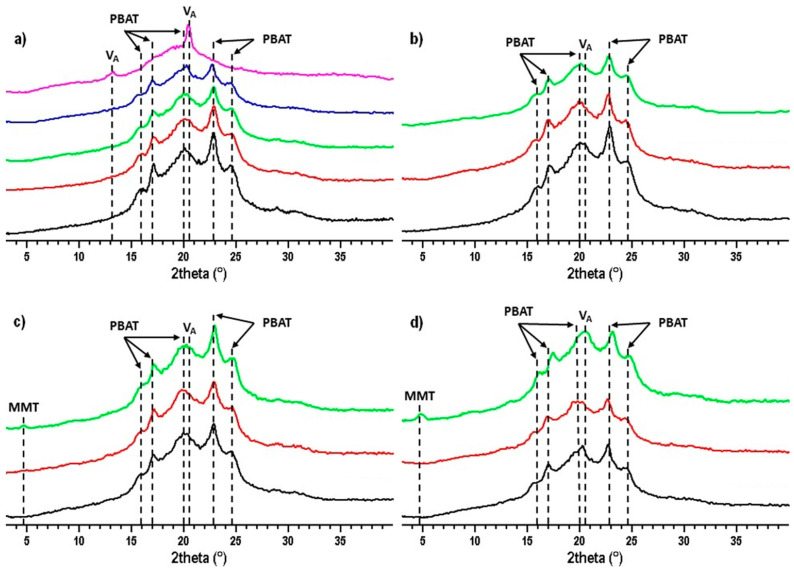
X-ray diffractogram of pure TPS (pink curve) and PBAT (black) and TPS/PBAT blends (10/90—red; 20/80—green; and 30/70—blue) without MMT (**a**) and TPS/PBAT blends with MMT content for blend ratios of 10/90 (**b**) 20/80 (**c**), and 30/70 (**d**). The contents of MMT are 0 (black curves), 2 (red), and 5 parts per 100 parts of starch (green).

XRD measurements were carried out to detect crystalline features in the sample structure and their changes during storage. Figure 1a shows the diffractogram of samples without nanofiller and pure TPS and PBAT samples. The PBAT diffractogram (Figure 1a; black curve) shows diffraction maxima at 2theta ≈ 16°, 17.1°, 20.1°, 22.8°, and 24.5°, which correspond to the crystalline domains of PBAT [41]. In the diffractogram of the TPS sample (Figure 1a; pink curve), a small peak at position 2theta ≈ 13.2° and a pronounced maximum at 2theta ≈ 20.4° appear corresponding to the TPS crystalline structures of the V_A_-type [42,43]. As the TPS content in the investigated blends increases, the peak for the V_A_-type TPS crystalline structures at 2theta ≈ 20.4° becomes more pronounced. Comparing the TPS/PBAT blends (Figure 1a; red, green, and blue curves), a decrease in the intensities of PBAT diffraction maxima are observed with decreasing PBAT content. Higher and narrower peaks at higher PBAT contents indicate better ordering of the PBAT crystalline domains since TPS can limit formation of crystalline domains in blends. The diffractograms for TPS/PBAT blends are more similar to the diffractogram of PBAT than TPS, which is due to the prevailing PBAT content.

Figure 1b shows diffractograms of a 10 TPS to 90 PBAT mixture without MMT (black curve) and with 2% MMT (red curve) and 5% MMT (green curve). Diffractograms do not differ much from each other. The addition of MMT nanoparticles (at low TPS content) has no significant effect on the crystalline phase in the blends.

Diffractograms of TPS/PBAT mixtures at a 20 to 80 ratio show almost identical diffractograms (Figure 1c) regardless of whether they are without MMT or containing 2 or 5 parts of MMT (related to the starch content). The sample containing 5 php of MMT was measured up to 4 days after preparation and may partially crystallize during that time. The fact that the maxima at 2theta ≈ 22.8° and 24.5° show higher intensities compared to the samples without MMT or containing 2 php of MMT supports this assumption. At the same time, a new diffraction maximum at position 2theta ≈ 4.8° corresponding to the intercalated MMT appears in the diffractogram of the sample with 5 php of MMT [44]. The fact that this maximum was not observed with 2 php MMT and in samples with a TPS/PBAT ratio 10 to 90 may indicate that MMT particles were exfoliated, or (more likely) that due to a low TPS/PBAT ratio and resulting low MMT content, XRD could not detect the MMT structure due to the insufficient size of crystalline structures [45].

A slightly different pattern is observed in the diffractogram of samples with a 30/70 TPS/PBAT ratio (Figure 1d). Similar to the 20/80 ratio sample, we also observe a diffraction maximum for the 2theta ≈ 4.8° position related to the intercalated MMT. This maximum is higher and broader compared to the 20/80 TPS/PBAT ratio with the 5 php of MMT sample, probably due to the higher MMT content in the blend.

### 3.2. Mechanical Properties

Standard mechanical properties are the most important data regarding any application of plastic-based materials. Generally, by the addition of TPS to PBAT, an increase in brittleness and a decrease in elongation at break can be expected. The ultimate mechanical properties of the TPS–MMT/PBAT nanocomposites are presented in Table 1. It is seen that the tensile strength and elongation at break of the blends exhibit a significant decrease with the addition and rising amount of TPS. This indication is ascribed to low tensile strength and elongation at break of TPS. Moreover, the increase in TPS content results in a monotonous increase in Young’s modulus, indicating the hard and brittle nature of the TPS [46]. The tendency of the modulus to increase is confirmed also by data from DMTA which were taken from curves of storage modulus (E′) at the temperature of 20 °C corresponding roughly to the conditions of tensile properties determination (Table 2).

Considering the incorporation of MMT, the tensile strength of the nanocomposites is decreasing by the addition of 2% MMT, while the tensile strength of the blends with 5% MMT are more or less the same as the samples containing 2% MMT. This observation can be explained by the formation of an exfoliated structure at higher MMT contents [47]. On the other hand, Young’s modulus is increasing with the increase in the MMT content. This increase in modulus may be caused by the increase in the number of exfoliated MMT sheets and, consequently, the reinforcing effect. As expected, the elongation at break of the blends decreased with the addition of MMT nanoparticles.

**Figure 2:**
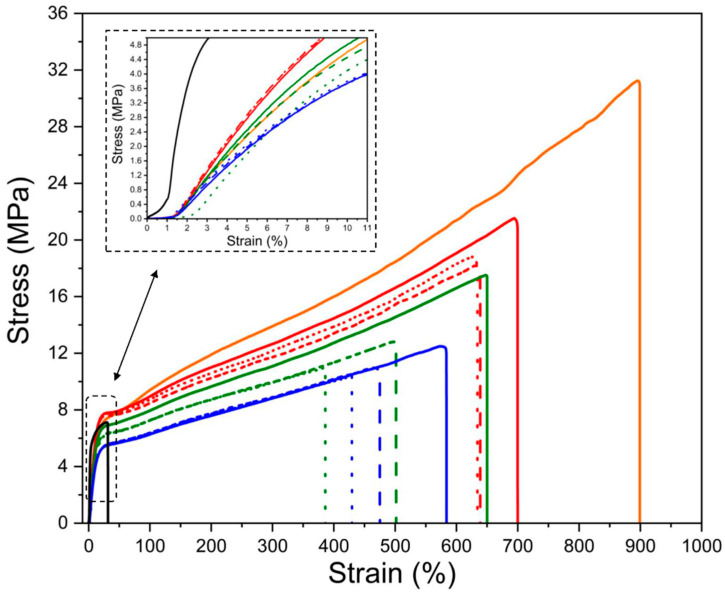
Stress–strain curves for the neat TPS (black line) and PBAT (orange), as well as TPS/PBAT blends of the ratios 10/90 (red), 20/80 (green), and 30/70 (blue). The content of MMT was 0 (full line ―), 2 (dashed line – – –), and 5 (dotted line **^…^**) parts per 100 parts of starch.

The more detailed analysis of the data in Table 1 is supported by Figure 2, where stress–strain curves are shown. Except for neat TPS, all curves have the same shape, without local maximum, indicating a yield point in the region after the initial Hookean-type of deformation (straight line at the beginning of stress–strain curve corresponding to the Hooke law stating that the force is proportional to the extension). This is somewhat unusual, especially for neat PBAT, which would be expected to behave similarly to polyethylene with a clear peak at the yield point.

Neat TPS behaves as a typical brittle material with a plasticizer content, exhibiting high Young’s modulus and low deformation at break. Mixtures of PBAT with TPS demonstrate a similar tendency, showing a decrease in elongation at break with rising TPS content. At the same time, the tensile strength also decreases, especially because of the lower elongation so that considering a similarity in the stress–strain curves, breaking the specimen at lower deformation, at break results also in lower tensile stress. However, considering the ratio ε/σ of elongation and tensile stress, it is seen that the data for a particular TPS/PBAT ratio ε/σ are the same when changing the MMT content from 0 to 5, but the ε/σ is rising if the composition of the polymers is increasing from TPS/PBAT 10/90 to 20/80, and 30/70 (ε/σ = 33 ± 0.3, 36.3 ± 1.1, and 43.7 ± 0.6, respectively). This indicates that the effect of the MMT addition is almost negligible on the tensile stress. Similar behavior is observed also for the addition of MMT to starch on Young’s modulus values. The effect is only marginal, if any. The reason may consist in the way of mixing, where the MMT is dispersed in the plasticizer before being mixed into the starch. By using such a method, it was expected that MMT particles would exfoliate to a greater extent but, obviously, at the same time, they created interactions with the plasticizer so that the surface of reinforcing MMT was be deactivated to certain extent.

### 3.3. Dynamic Mechanical Thermal Analysis (DMTA)

DMA analysis detects temperature-induced transitions in the investigated materials demonstrated as a distinct decrease in the temperature dependence of storage modulus E′, representing stored energy in one cycle of sample deformation, and as local maxima in the temperature dependences of the loss modulus E″ determining the dissipated energy in one cycle of sample deformation and the loss factor tanδ equal to E″/E′.

Relaxation transitions in polymers are associated with an increase in the molecular mobility. When the polymer system is in the glassy state, only vibrational and rotational motions of atoms and short segments can take place, while with increasing temperature, adjacent group motion is activated, then segmental motion is allowed, and at a sufficiently high temperature, a large-scale chain slippage occurs. During relaxation transitions, the energy is dissipated into the polymer system which results in thermal motion accompanied by the decrease in storage modulus and local maxima in the tanδ temperature dependence indicating a significant rearrangement of the polymer chains [48,49].

**Figure 3:**
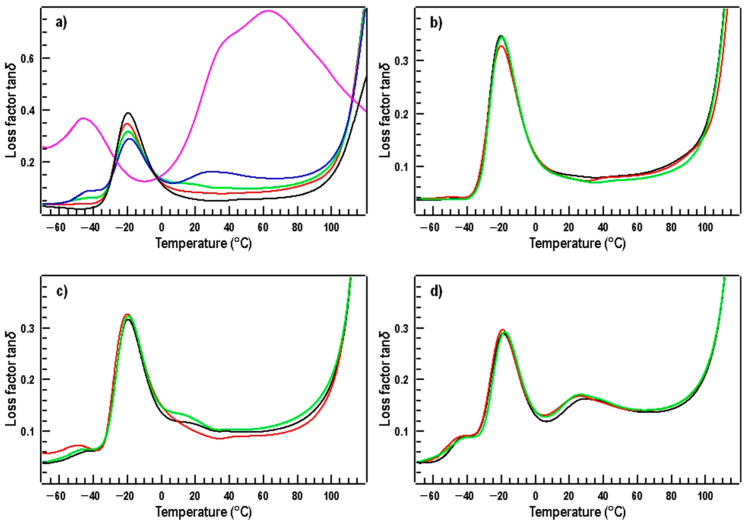
Temperature dependences of tan δ for pure TPS (pink curve) and PBAT (black) and TPS/PBAT blends (10/90—red; 20/80—green; and 30/70—blue) without MMT (**a**), and TPS/PBAT blends with MMT content for blend ratios of 10/90 (**b**), 20/80 (**c**), and 30/70 (**d**). The contents of MMT are 0 (black curves), 2 (red), and 5 parts per 100 parts of starch (green).

The loss factor tanδ temperature dependence of the PBAT sample (Figure 3a; black curve) shows a maximum at −19.6 °C [50], corresponding to a glass transition in the amorphous PBAT domains. Below this temperature, the movement of the macromolecule segments consists only in vibration or rotation, while at temperatures above T_g_, the segments can also change their position. Therefore, the polymeric material changes from a hard and brittle substance (in the glassy state) to a highly elastic one (in the rubbery state) as movement of the chain segments is promoted with the temperature increase. Another relaxation transition is observed above 120 °C when overall chain mobility is enhanced due to melting of PBAT crystalline domains. In the case of the TPS sample (Figure 3a; pink curve), two distinct maxima related to the glass transitions in the plasticizer (glycerol)-rich (at −46 °C) and in the starch-rich regions (above 0 °C) are evident. Two separate relaxation transitions, present within the broad maximum above 0 °C, can be assigned to segmental motion of the linear amylose chains (at 40 °C) and the branched amylopectin chains (at 63 °C) [51,52]. In these domains, segmental motion of the amylose and amylopectin chains take place, respectively. The glass transition temperature for the segmental motion of amylose chains is observed (usually) at lower temperatures than in the case of amylopectin. If we observe only one wide maximum for starch-rich domains on the temperature dependence of the loss factor tanδ, this may indicate a possible overlap of the glass transitions of the segmental motion of the amylose and amylopectin chains [52]. We see such a possible scenario in the graphs of Figure 3a,c,d. The most pronounced maximum for starch-rich domains is for blends with a 30/70 TPS/PBAT ratio (Figure 3d). In general, PBAT and TPS are immiscible polymers, and therefore, four relaxation transitions can be expected for the TPS/PBAT blends. The loss factor tanδ temperature dependence of all investigated blends (Figure 3a–d) shows a maximum around −20 °C, corresponding to glass transition in amorphous PBAT domains. Differences in the glass transition temperatures for PBAT in TPS/PBAT blends are in the range of ±1 °C, which is within the scatter of the measurements. In the case of TPS/PBAT blends, melting at lower temperatures was observed compared to the virgin PBAT sample, probably due to the continuous PBAT matrix being distorted by the presence of the TPS domains.

Comparing the blends with different TPS/PBAT ratios (Figure 3a), two relaxation transitions (at −41 °C and 30 °C) can be clearly seen for the blend of a TPS/PBAT ratio of 30/70 (Figure 3a; blue curve), related to glycerol-rich and starch-rich regions, respectively. These maxima are less pronounced and shifted to slightly lower temperatures (at −44 °C and 20 °C) for the ratio of 20/80 (Figure 3a; green curve). This indicates a slightly higher mobility of the chains in the TPS domains. Relaxation transitions in TPS domains are not seen at the temperature dependence on the loss factor tanδ for the TPS/PBAT ratio of 10 to 90 (Figure 3a, red curve) due to the lower TPS content.

By comparing the temperature dependences of TPS/PBAT blends (Figure 3b–d) with the same TPS/PBAT ratio (10/90, 20/80 and 30/70) and different MMT content, it is seen that the curves are (almost) identical. In the case of blends with a ratio of 10/90 (Figure 3b), only glass transition and melting of PBAT chains are observed due to the low TPS content. In the case of the blends with a 20/80 TPS/PBAT ratio (Figure 3c), glass transition for glycerol-rich domains and starch-rich domains can already be observed. As the MMT content increases, minor changes also occur. The glass transition temperature for starch-rich domains decrease from about 20 °C to 15 °C for a TPS/PBAT ratio of 20/80 without MMT and with 5 php MMT, respectively. For TPS containing 2 php MMT, the glass transition temperature cannot be accurately determined. The glass transition temperature for the glycerol-rich regions also decrease to lower temperatures. The temperature dropped from −42 °C, through −45 °C to −49 °C for rising MMT content in the TPS/PBAT mixture of 20/80. A similar trend as in the case of the blends with a TPS/PBAT ratio of 20/80 (Figure 3c) is also observed for the blends with a TPS/PBAT ratio of 30/70 (Figure 3d), where the glass transition temperature of starch-rich domains decreased from 30 °C to 26 °C for the samples with 0% MMT and 5% MMT, respectively. The changes in glass transition temperature for the glycerol-rich regions were small (±2 °C). These transitions took place at about −42 °C. The lowering of the glass transition temperature is related to the higher mobility of starch chains.

**Figure 4:**
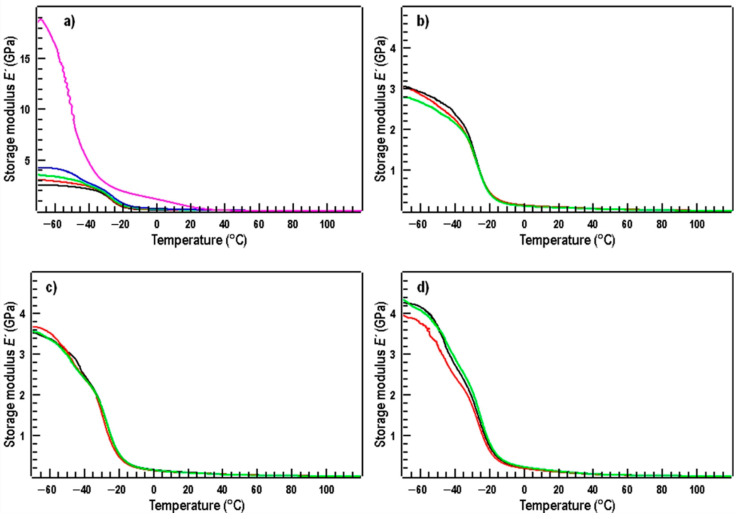
Temperature dependences of storage modulus for pure TPS (pink curve) and PBAT (black) and TPS/PBAT blends (10/90—red; 20/80—green; and 30/70—blue) without MMT (**a**), and TPS/PBAT blends with MMT content for blend ratio 10/90 (**b**), 20/80 (**c**), and 30/70 (**d**). The contents of MMT are 0 (black curves), 2 (red), and 5 parts per 100 parts of starch (green).

In Figure 4, the temperature dependences of the storage modulus E′ of all the samples are shown. By comparing the samples within and between graphs, it can be concluded that the TPS sample appears to be the stiffest material. This corresponds to the high values of storage modulus at negative temperatures as well as the value of the storage modulus at room temperature (Table 2). Samples with the same TPS/PBAT ratio do not differ much in the temperature dependences and values of storage modulus at room temperature.

### 3.4. Nuclear Magnetic Resonance (NMR)

**Figure 5:**
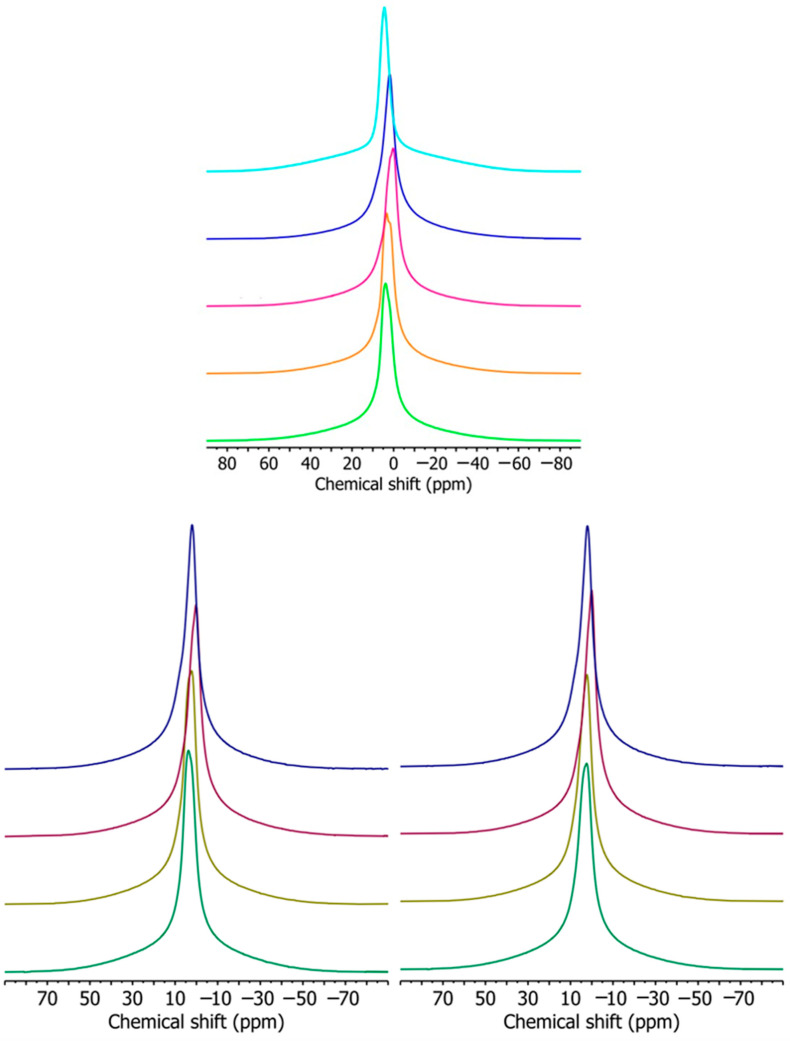
Figure on the top, ^1^H BL NMR spectra of pure TPS (light blue), PBAT (dark blue), and the blends of TPS/PBAT ratios 10/90 (magenta), 20/80 (orange), and 30/70 (green). Figure on the bottom, ^1^H BL NMR spectra of samples TPS/PBAT containing 2 (left) and 5 (right) parts of MMT per 100 parts of starch. The samples from top to bottom: neat PBAT (dark blue), TPS/PBAT ratios 10/90 (purple), 20/80 (olive), and 30/70 (dark green).

The widths of signals in ^1^H BL NMR spectra provide information on the molecular mobility in polymer materials since ^1^H nuclei in rigid and mobile chains produce broad and narrow signals, respectively [53]. The ^1^H BL NMR spectra of studied samples (Figure 5) consist of one broad (BS) and one narrow signal (NS). The broad signal originates from ^1^H nuclei in rigid starch chains, immobile PBAT chains, and bound water. The narrow signal comes from the ^1^H nuclei in more mobile glycerol molecules, free water molecules (for samples containing TPS), and more mobile PBAT chains (for samples containing PBAT). The apparent chemical shift of this signal ranges from 4.2 to 2.1 ppm. With the changing TPS/PBAT ratio (Figure 5) in the samples, the chemical shift of the narrow signal also changes.

The narrow signal of the TPS/PBAT blends is a superposition of the two signals—one from ^1^H nuclei in CH_2_ groups in PBAT at a chemical shift of 1.7 ppm (NS1) and one from ^1^H nuclei in glycerol, water, and OCH_2_ groups in PBAT at a chemical shift of 4.4 ppm (NS2) [54].

**Figure 6:**
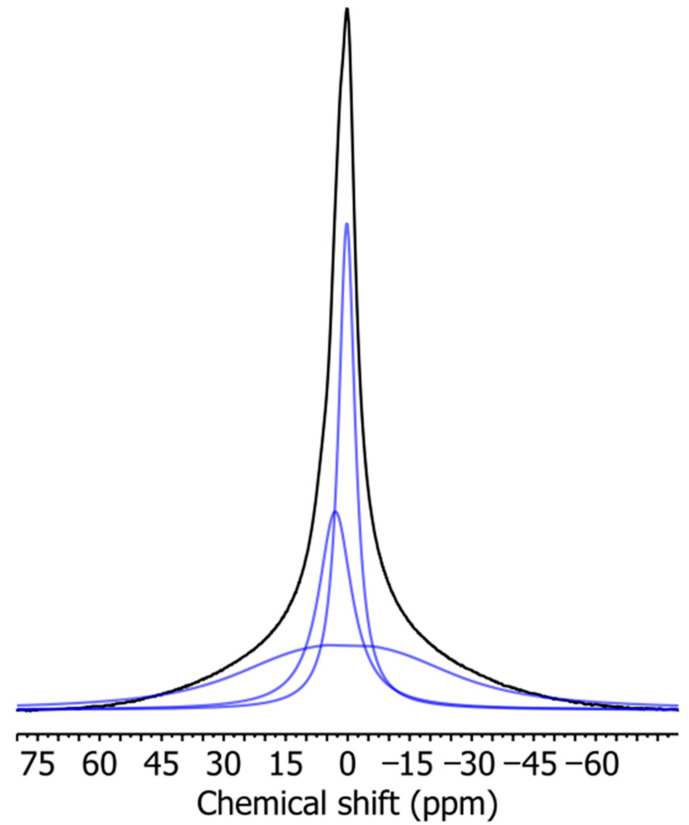
Deconvolution of ^1^H BL NMR spectrum of the sample TPS/PBAT ratio of 10/90, with addition of 2 parts of MMT per 100 parts of starch. Black curve represents the measured spectrum, and blue curves are the curves obtained by the deconvolution of the measured spectra to individual processes, described in the discussion part.

**Figure 7:**
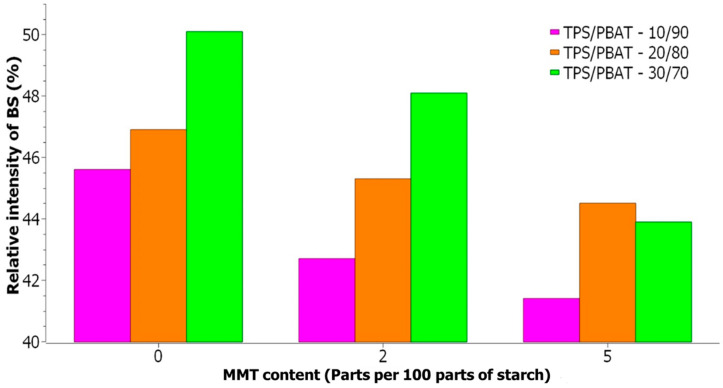
Relative intensity of broad signal (BS) in ^1^H BL NMR spectra for mixtures TPS/PBAT with varying content of MMT in php (parts per 100 parts of starch); ratios of the components are shown inside the figure.

**Figure 8:**
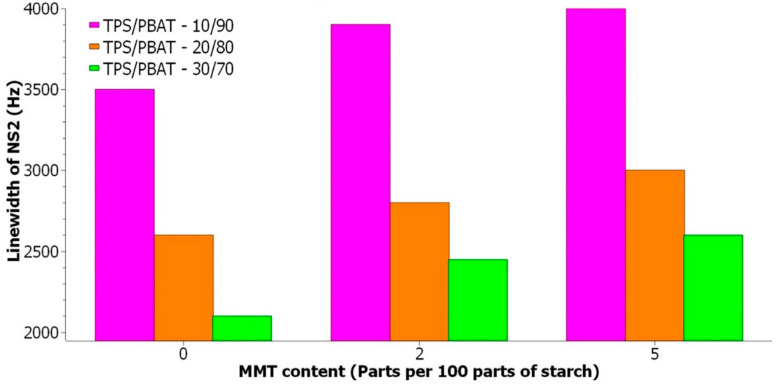
Linewidth of narrow signal NS2 in ^1^H BL NMR spectra for mixtures of TPS/PBAT with varying content of MMT in php (parts per 100 parts of starch); the composition of TPS/PBAT ratios are shown inside the figure.

By deconvolution of ^1^H BL NMR spectra (Figure 6), it is possible to obtain information about the width and intensity of individual signals (Table 3). With increasing TPS content in the studied samples, the relative intensity of the broad signal increases (Figure 7), which indicates an increase in the rigid fraction in TPS/PBAT blends. However, the line width of NS2 signals decreases (Figure 8) which points to the enhanced mobility of water and glycerol molecules in TPS domains probably due to the hydrophobic nature of the PBAT matrix. This effect is more pronounced for samples containing MMT, and it may be related to the size of TPS domains since larger TPS domains provide better conditions for the motion of water and glycerol molecules.

With the increase in MMT content in the samples with particular TPS content, the relative intensity of the broad signal decreases (Figure 7), which indicates the increasing number of intercalated species in the MMT interlayer space which are less mobile than those that are free but more mobile than those that could be entrapped in the TPS structure [29]. The width of NS2 signals for samples with a particular TPS content increases with the increasing MMT content (Figure 8), indicating decreased molecular mobility due to the interaction of relevant species with MMT layers.

**Figure 9:**
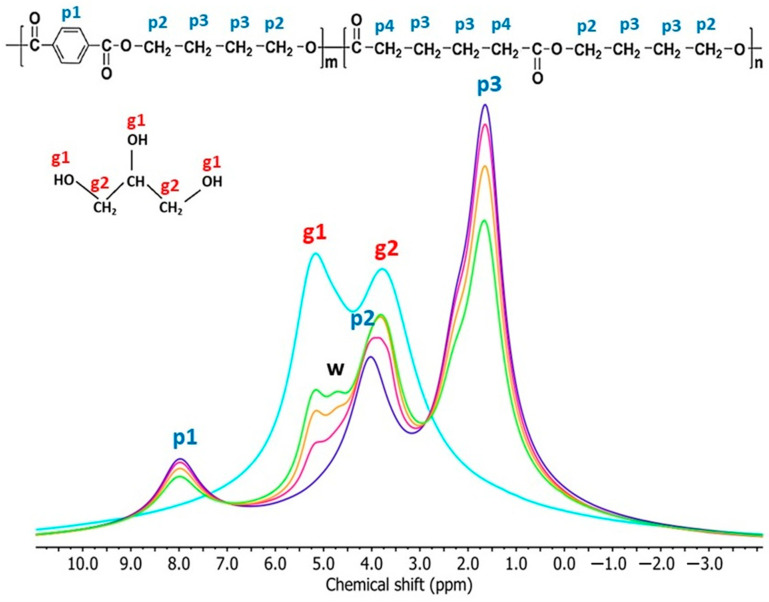
^1^H MAS NMR spectra of studied samples, showing signals for particular hydrogens. Composition of polymeric matrices: TPS—light blue; PBAT—dark blue; TPS/PBAT 10/90—magenta; 20/80—orange; 30/70—green.

^1^H MAS NMR spectrum of the TPS sample consists of two well-resolved signals with chemical shift values of 5.2 ppm and 3.7 ppm from the ^1^H nuclei in glycerol (Figure 9). The signal from the ^1^H nuclei in the starch molecules is found in the broad signal and in the rotational signals (not shown in Figure 9). The ^1^H MAS NMR spectrum of the PBAT sample contains three signals with chemical shift values of 8.0 ppm, 4.0 ppm, and 1.6 ppm, which originate from the ^1^H nuclei in the terephthalate unit, OCH_2_, and CH_2_/CH group in PBAT, respectively. The signal with a chemical shift of 1.6 ppm is a superposition of three signals from p4, p5, and p6 hydrogens. In the ^1^H MAS NMR spectra of TPS/PBAT blends, there are four signals, with chemical shift values of 8.0 ppm, 5.2 ppm, 3.8 ppm, and 1.6 ppm, which originate from the ^1^H nuclei in PBAT and glycerol within the TPS component [55]. The signal with a chemical shift value of 3.8 ppm is a superposition of signals from p2, p3 PBAT hydrogen nuclei, and ^1^H nuclei in CH_2_/CH groups in glycerol. In these spectra, another signal with a chemical shift value of 4.7 ppm is observed, which is attributed to ^1^H nuclei in mobile water molecules [45,54]. This signal is not observed in the spectrum of the TPS sample. It may mean that the hydrophobic PBAT matrix surrounding TPS domains is able to displace water molecules located in the TPS domains of TPS/PBAT blends and thereby increases their molecular mobility.

**Figure 10:**
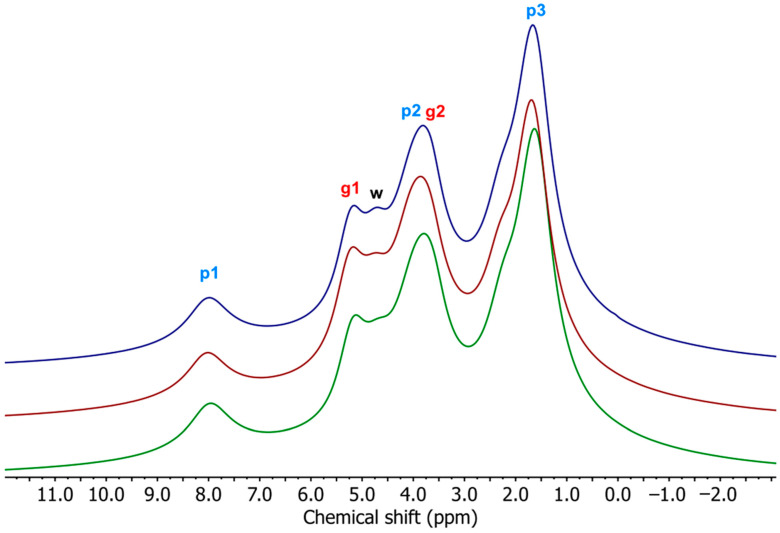
^1^H MAS NMR spectra for samples of TPS/PBAT with ratios of 30/70 and 0, 2, and 5 parts of MMT per 100 parts of starch (php). The samples from top to bottom: 0 (dark blue), 2 (purple), and 5 (green) php of MMT.

The ^1^H MAS NMR spectra of TPS/PBAT blends containing MMT do not differ much from those for samples without MMT (Figure 9). With increasing MMT content, a small difference is seen in decreasing the intensity of the mobile water signal with a chemical shift of 4.7 ppm, which is the most conspicuous for samples with the highest TPS content (Figure 10), due to the lower mobility of water molecules, as was detected in BL spectra as the NS2 linewidth increases (Figure 8).

**Figure 11:**
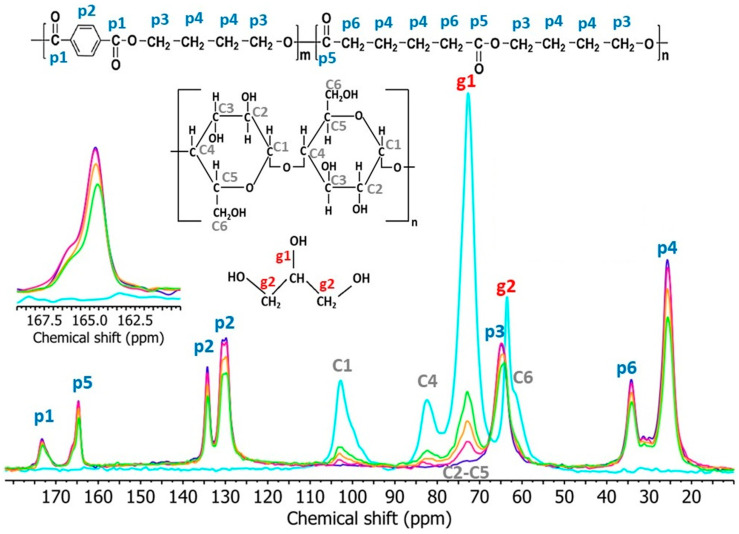
^13^C CP/MAS NMR spectra of TPS—light blue; PBAT—dark blue; TPS/PBAT 10/90—magenta; 20/80—orange; 30/70—green.

Figure 11 shows the measured ^13^C CP/MAS NMR spectra of TPS, PBAT, and TPS/PBAT blends. The chemical shifts of the individual signals and the functional groups from which they originate are listed in Table 4, assigned in Figure 11 according to [56,57]. The resonance from the C1 carbons in starch in the spectra for TPS and TPS/PBAT samples shows only one broad signal with an amplitude at the 103 ppm chemical shift. The shape of this signal indicates that TPS chains are disordered in the studied samples. The signal from C6 carbons in starch is not observed in the spectra for TPS/PBAT blends probably due to a low intensity, a large line width, and an overlap with the p3 signal of the PBAT component [58].

**Figure 12:**
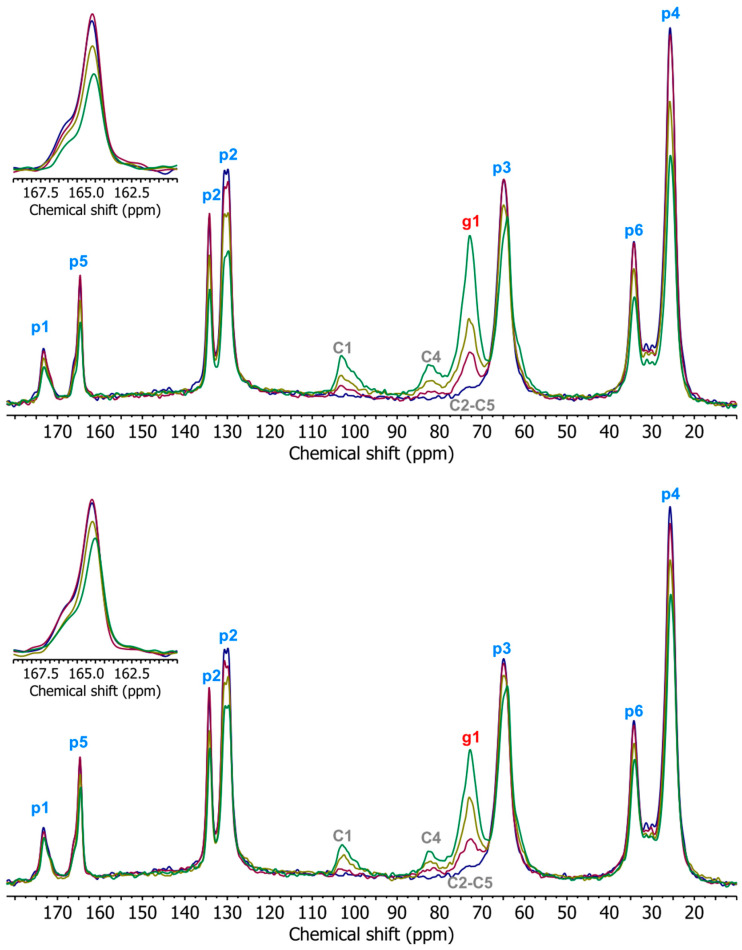
^13^C CP/MAS NMR spectra of studied samples, showing signals for particular carbon atoms. Figure on the top, MMT content 2 php (parts per 100 parts of starch); on the bottom, MMT content 5 php in each sample; composition of polymeric matrices: PBAT—dark blue; TPS/PBAT 10/90—purple; 20/80—olive; 30/70—green.

The presence of TPS in studied blends affects the molecular mobility of PBAT chains. This effect can be deduced from the comparison of the spectra for PBAT and TPS/PBAT 10/90 (Figure 11). The intensities of signals from p5 in the spectrum of the TPS/PBAT 10/90 blend and for neat PBAT are almost the same (despite the lower content of PBAT in the sample). The same effect is observed for signals of p1, p3, and p6 carbons of PBAT and also for blends containing MMT (Figure 12) This may be due to the fact that signals from less mobile parts in the sample are enhanced in ^1^H-^13^C CP/MAS NMR spectra. The presence of 10 wt% of TPS in the sample decreases the mobility of the PBAT chains, mainly those located at the TPS/PBAT interfaces.

## 4. Conclusions

The investigation of mixtures of PBAT with thermoplastic starch indicates that TPS addition results in a substantial decrease in tensile strength and an increase in the brittleness of the blends with rising TPS content. This tendency is even more visible after the addition of montmorillonite particles as the reinforcing filler indicated by the significant increase in the Young’s modulus and further decrease in the elongation at break. However, even the addition of 30% of TPS results in maintaining sufficiently high values of elongation at break, i.e., the blend is still quite tough in spite of the presence of 30% highly brittle TPS.

DMTA results reveal the formation of TPS domains in the PBAT matrix. The two relaxation transitions correspond to a presence of glycerol-rich and starch-rich regions, respectively. These conclusions are convincingly supported by the NMR measurement of chain mobility, which is higher in the glycerol-rich compared to starch-rich regions.

The effect of the MMT addition is almost negligible on the tensile stress of the blends due to interactions between MMT and plasticizer molecules. The reason is seen in the way of the mixture preparation where the MMT is dispersed in the plasticizer before mixing into the starch, so that the reinforcing effect of the MMT nanoparticles through the interactions of MMT with starch macromolecules are not prevailing; although, MMT has been intercalated and partially exfoliated according to XRD measurements supported by mechanical parameters evaluation.

The presented data suggest the possibility of applications of the mixtures of PBAT with TPS up to 20 and in some cases 30 wt% for less demanding applications, which makes the price of the biodegradable plastics material competitive with common polyolefins, if accepting a certain decrease in mechanical parameters of the final mixture.

All conclusions mentioned above are fully supported by measurements of the polymer chain mobilities performed with NMR, making the conclusions much more understandable and reliable.

## Figures and Tables

**Table 1:** Mechanical properties of the TPS–MMT/PBAT nanocomposites depend on the blend composition; TPS—thermoplastic starch; PBAT—Poly(butylene adipate–co–terephthalate); MMT—montmorillonite.

TPS wt%	PBAT wt%	MMT php *	Tensile Stress (MPa)	Tensile Strain (%)	Young’s Modulus (MPa)
0	100	0	29.6 ± 3.3	845 ± 96	42.8 ± 3.4
10	90	0	21.3 ± 0.9	696 ± 44	68.7 ± 2.9
10	90	2	18.7 ± 1.4	627 ± 57	76.0 ± 2.6
10	90	5	18.8 ± 0.8	629 ± 35	73.0 ± 5.0
20	80	0	17.2 ± 1.8	641 ± 58	63.3 ± 1.4
20	80	2	13.0 ± 2.1	500 ± 101	59.6 ± 4.1
20	80	5	11.2 ± 2.0	380 ± 134	63.9 ± 1.3
30	70	0	13.1 ± 1.4	586 ± 61	50.2 ± 2.0
30	70	2	10.7 ± 2.0	469 ± 128	52.6 ± 3.7
30	70	5	10.3 ± 1.5	429 ± 100	53.0 ± 2.0
100	0	0	7.2 ± 0.5	33.7 ± 8.6	363 ± 20

* php—parts per hundred parts of starch.

**Table 2:** Storage modulus values (GPa) at room temperature (20 °C) of the TPS–MMT/PBAT nanocomposites depend on the blend composition; TPS—thermoplastic starch; PBAT—Poly(butylene adipate–co–terephthalate); MMT—montmorillonite.

TPS wt%	PBAT wt%	MMT php *	Storage Modulus (GPa)
0	100	0	70
10	90	0	84.7
10	90	2	82.3
10	90	5	78.3
20	80	0	94.2
20	80	2	85.3
20	80	5	85.8
30	70	0	125
30	70	2	103.7
30	70	5	123.2
100	0	0	395

* php—parts per hundred parts of starch.

**Table 3:** Deconvolution of ^1^H BL NMR spectra of studied PBAT/TPS mixtures into broad (BS) and two narrow signals (NS1 and NS2) as seen in Figure 6, dependent on the composition of blend components, thermoplastic starch (TPS, wt%), poly(butylene adipate–co–terephthalate) (PBAT, wt%), and montmorillonite (MMT, parts per hundred parts of starch) are given.

			NS1		NS2		BS	
TPS wt%	PBAT wt%	MMT php *	Linewidth (Hz)	Rel. Int. (%)	Linewidth (Hz)	Rel. Int. (%)	Linewidth (Hz)	Rel. Int. (%)
0	100	0	1700	27.2	3100	22.7	17,000	50.1
10	90	0	1900	28.3	3500	26.1	22,000	45.6
10	90	2	1950	31.6	3900	25.7	22,500	42.7
10	90	5	2050	34.6	4000	24	23,000	41.4
20	80	0	1700	19.5	2600	33.6	20,000	46.9
20	80	2	1800	21.8	2800	32.9	21,000	45.3
20	80	5	1900	25.7	3000	29.8	22,500	44.5
30	70	0	1900	17.6	2100	32.3	21,000	50.1
30	70	2	2150	21.8	2450	30.1	22,000	48.1
30	70	5	2200	26.2	2600	29.9	23,000	43.9
100	0	0	1900	60.2	-	-	26,000	39.8

* php—parts per hundred parts of starch.

**Table 4:** Overview of chemical shifts in ^13^C CP/MAS NMR spectra of studied samples.

Component	Functional Group	Chemical Shift (ppm)
PBAT	–COOR (p1, p5)	173.2
		164.6
	–C_6_H_4_– (p2)	134.2
		129.6
	–CH_2_–COOR (p6)	34.2
	–CH_2_– (p4)	25.9
	–O–CH_2_ (p3)	65.2
TPS	C1	103
	C2–C5	65.5–78.8
	C4	82.1
	C6	61.4
Glycerol	CH_2_ (g2)	63.6
	CH (g1)	73

## Data Availability

The datasets presented in this article are not readily available because the data are part of an ongoing study.
